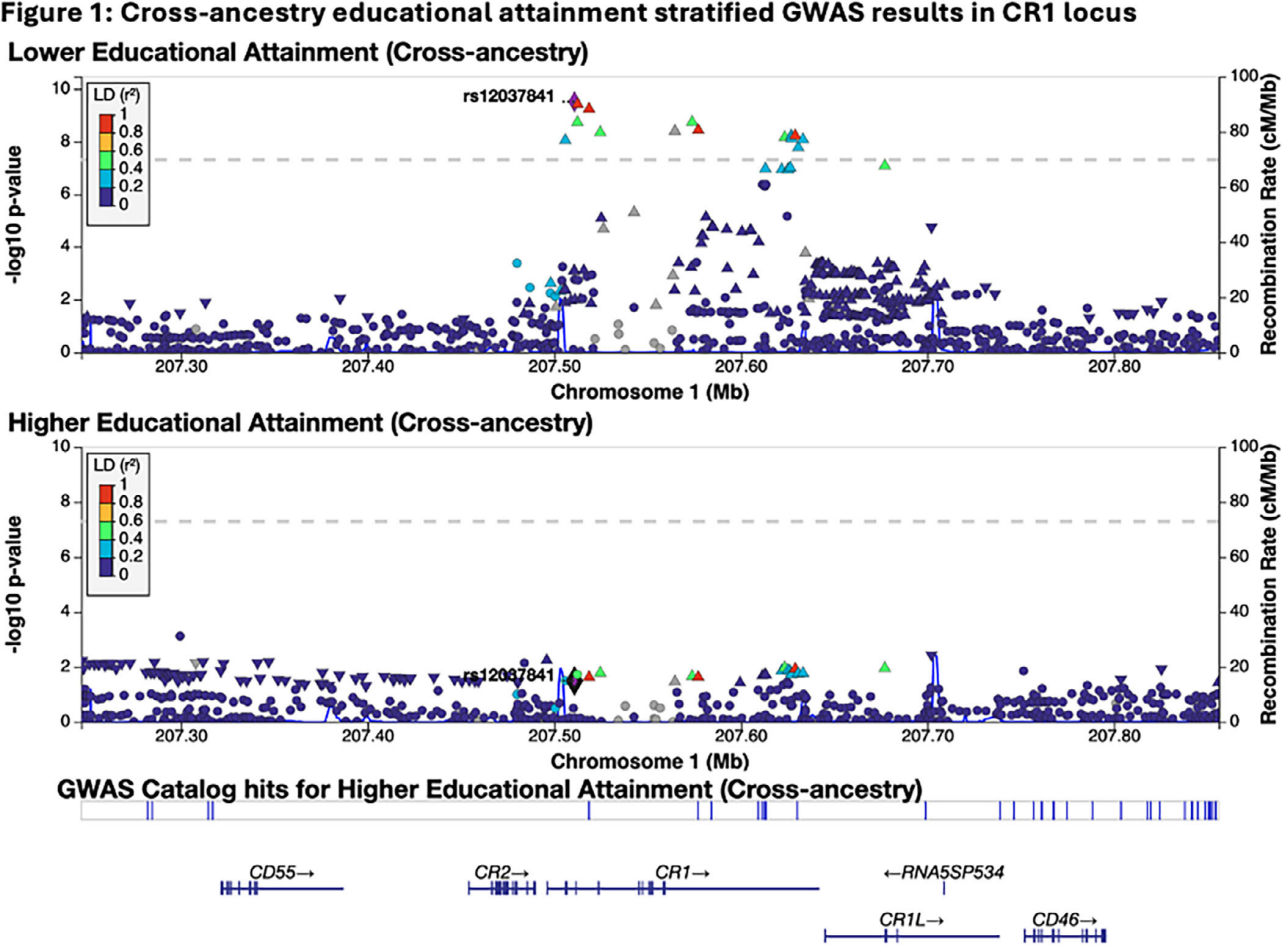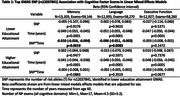# Genome‐wide interaction and stratified study reveals *CR1*‐Alzheimer's disease association is moderated by education level

**DOI:** 10.1002/alz70855_105938

**Published:** 2025-12-24

**Authors:** Ryan Dacey, Xudong Han, Shruti Durape, Jaeyoon Chung, Max Rosenthaler, Bobak Abdolmohammadi, Annie J. Lee, Adam Brickman, Timothy J. Hohman, Michael L Cuccaro, David A. A. Bennett, Andrew J. Saykin, Emily H. Trittschuh, Shubhabrata Mukherjee, Paul K Crane, M. Ilyas Kamboh, Walter W. Kukull, Rhoda Au, Jonathan L Haines, Margaret Pericak‐Vance, Gerald D. Schellenberg, Richard Mayeux, Kathryn L. Lunetta, Lindsay A. Farrer, Jesse Mez

**Affiliations:** ^1^ Department of Medicine (Biomedical Genetics), Boston University Chobanian & Avedisian School of Medicine, Boston, MA, USA; ^2^ Framingham Heart Study, Boston University Chobanian & Avedisian School of Medicine, Boston, MA, USA; ^3^ Boston University Alzheimer's Disease Research Center, Boston, MA, USA; ^4^ Boston University Chobanian & Avedisian School of Medicine, Boston, MA, USA; ^5^ Department of Neurology, Boston University Chobanian & Avedisian School of Medicine, Boston, MA, USA; ^6^ Boston University Alzheimer's Disease Research Center, Boston University Chobanian & Avedisian School of Medicine, Boston, MA, USA; ^7^ Department of Neurology, Taub Institute for Research on Alzheimer's Disease and the Aging Brain, Columbia University, New York, NY, USA; ^8^ Vanderbilt Memory and Alzheimer's Center, Vanderbilt University School of Medicine, Nashville, TN, USA; ^9^ Vanderbilt Genetics Institute, Vanderbilt University Medical Center, Nashville, TN, USA; ^10^ The John P. Hussman Institute for Human Genomics, University of Miami, Miami, FL, USA; ^11^ Department of Neurological Sciences, Rush Medical College, Chicago, IL, USA; ^12^ Department of Medical and Molecular Genetics, Indiana University School of Medicine, Indianapolis, IN, USA; ^13^ Indiana Alzheimer's Disease Research Center, Indiana University School of Medicine, Indianapolis, IN, USA; ^14^ Department of Radiology and Imaging Sciences, Indiana University School of Medicine, Indianapolis, IN, USA; ^15^ Geriatric Research, Education, and Clinical Center, Veterans Affairs Puget Sound Health Care System, Seattle, WA, USA; ^16^ Department of Psychiatry and Behavioral Sciences, University of Washington School of Medicine, Seattle, WA, USA; ^17^ Department of Medicine, University of Washington, Seattle, WA, USA; ^18^ Department of General Internal Medicine, University of Washington School of Medicine, Seattle, WA, USA; ^19^ Department of Human Genetics, University of Pittsburgh, Pittsburgh, PA, USA; ^20^ Biomedical Genetics, Department of Medicine, Boston University Medical School, Boston, MA, USA; ^21^ Department of Epidemiology, Boston University School of Public Health, Boston, MA, USA; ^22^ Department of Anatomy & Neurobiology, Boston University Chobanian & Avedisian School of Medicine, Boston, MA, USA; ^23^ Department of Population & Quantitative Health Sciences, School of Medicine, Case Western Reserve University, Cleveland, OH, USA; ^24^ Department of Pathology and Laboratory Medicine, University of Pennsylvania, Philadelphia, PA, USA; ^25^ Department of Biostatistics, Boston University School of Public Health, Boston, MA, USA; ^26^ Department of Neurology and Ophthalmology, Boston University Chobanian & Avedisian School of Medicine, Boston, MA, USA

## Abstract

**Background:**

Genetic and environmental factors contribute to Alzheimer's disease (AD) risk. Understanding gene‐environment interactions may provide insight into unexplained AD heritability. Higher educational attainment is associated with lower AD risk, but the mechanism remains unclear. We conducted a genome‐wide association study (GWAS) to explore genetic‐education‐related associations with AD through SNP‐education interaction and education‐stratified analyses.

**Method:**

Educational attainment data were available and analyzed among 23,642 non‐Hispanic white (NHW; 10,272 cases) and 3,461 African American (AFA; 1064 cases) participants from the AD Genetic Consortium and the Framingham Heart Study. Educational attainment was dichotomized by median years of education across cohorts, which equated to completing four years of college. Across 35 datasets, we conducted separate GWAS: 1) including a SNP‐by‐education interaction term and 2) stratifying by median education status. MAGEE was used to estimate SNP‐by‐education interaction effects and SAIGE was used to estimate SNP effects in stratified analysis. GWAS models adjusted for age, sex, and principal components for population structure. METAL was used for inverse‐variance weighted within‐ancestry fixed‐effects meta‐analysis and METASOFT was used to estimate cross‐ancestry effects. Top GWAS hits were further analyzed for association with longitudinal trajectories of harmonized memory, language, and executive function factor scores in education‐stratified linear mixed effects models.

**Result:**

Stratified GWAS identified a genome‐wide significant association among participants with lower educational attainment in *CR1*, a well‐known AD‐associated locus on chromosome 1 (top SNP: rs12037841; lower educational attainment: MAF=0.19, OR=1.33, *p* = 3.1x10^‐10^; higher educational attainment: MAF=0.19, OR=1.09, *p* = 0.03; interaction‐model: β_snpXedu_=‐0.18, *p* = 0.0018). Effects among those with lower educational attainment were present in both ancestries (NHW: MAF=0.19, OR=1.30, *p* = 1.8x10^‐9^; AFA: MAF=0.03, OR=1.64, *p* = 0.02). In analysis with neuropsychological factor scores, rs12037841 was associated with faster decline in memory and language among participants with lower educational attainment (memory: β_snpXtime_=‐0.010, 95% CI:[‐0.016,‐0.004], *p* = 0.0019; language: β_snpXtime_=‐0.006, 95% CI:[‐0.011,‐0.002], *p* = 0.0083). Weaker, non‐significant effects were observed among participants with higher educational attainment.

**Conclusion:**

In educational attainment‐stratified GWAS of AD, we identified stronger association of known AD‐related gene *CR1*, among those with lower educational attainment. The finding implicating *CR1*, a complement pathway gene, suggests that the risk education confers on AD may be moderated by immune‐related mechanisms.